# Do illegitimate tasks matter for registered nurses’ work motivation? A cross-sectional study based on a nationally representative sample of Swedish nurses

**DOI:** 10.1016/j.ijnsa.2023.100159

**Published:** 2023-10-14

**Authors:** Carina Ahlstedt, Linda Moberg, Emma Brulin, Anna Nyberg

**Affiliations:** a Department of Public Health and Caring Sciences, Health Services Research, Uppsala University, Sweden; b Department of Government, Uppsala University, Sweden; c Institute of environmental medicine, Unite of Occupational medicine, Karolinska Institutet, Sweden; d Department of Public Health and Caring Science, Health Equity and Working Life, Uppsala University, Sweden

**Keywords:** Cross-sectional survey, Healthcare organisations, Motivation, Registered nurses, Work engagement

## Abstract

**Background:**

A challenge in Western countries is the growing need for registered nurses (RNs’) in hospitals, primary care and home healthcare. Decreasing illegitimate tasks and strengthening RNs’ work motivation are some strategies to address this challenge.

**Objective:**

Our overall aim was to explore the association between RNs' experiences of illegitimate tasks and work motivation operationalised as four dimensions: work engagement, opportunities to provide high-quality care, employer satisfaction and intention to remain at the workplace. To address this aim, three specific research questions were asked: (1) Is there an association between illegitimate tasks and work motivation? (2) Do the levels of reported illegitimate tasks differ between RNs working in hospitals and those working in primary care or home healthcare settings? (3) Do associations between illegitimate work tasks and work motivation differ with type of workplace?

**Design:**

A cross-sectional design.

**Methods:**

We used responses from a stratified population of RNs in Sweden, *n* = 2,333, working either in hospitals, primary care or home healthcare. Calibrating weights were applied in all analyses to ascertain the generalisability of the findings. Illegitimate tasks were measured with the Bern Illegitimate Tasks Scale. Data were analysed using chi-squared tests and linear or logistic regression analysis. Interaction was measured on the multiplicative scale by adding an interaction term to the fully adjusted models.

**Results:**

Overall, approximately 25 % of RNs reported frequently experiencing illegitimate tasks. There were statistically significant associations between higher perceptions of illegitimate tasks and lower ratings in the four dimensions of work motivation: work engagement [beta coefficient [beta] = -0.14, confidence interval [CI] 95 % = -0.18; -0.10], opportunities to provide high-quality care [beta = -0.46, CI 95 % = -0.51; -40] and employer satisfaction [beta = -0.60, CI 95 % = -0.67; -0.54]. Experiencing higher levels of illegitimate tasks also related to a decreased intention to remain at the workplace [illegitimate tasks: odds ratio = 0.32, CI 95 % = 0.27; 0.29]. RNs who worked in home healthcare reported higher levels of illegitimate tasks than RNs who worked in hospitals.

**Conclusions:**

Reducing the amount of illegitimate tasks may contribute to counteracting the shortage of RNs by increasing work motivation and willingness to remain at the workplace.

## What is already known

• Illegitimate tasks are a stressor and may affect health and emotions, causing for example lower self-esteem, poorer sleep, burnout and higher incidence of musculoskeletal pain.
• Work motivation can be increased if employees are assigned tasks that are meaningful.

## What this paper adds

• Registered nurses who experienced higher levels of illegitimate tasks reported lower levels of work motivation in terms of work engagement, opportunities to provide high-quality care, employer satisfaction and intention to remain at the workplace.
• Registered nurses who worked in home healthcare reported higher levels of illegitimate tasks than registered nurses who worked in hospitals.

## 1 Introduction

The World Health Organization (WHO) has identified the shortage of healthcare workers, including registered nurses (RNs), as a ‘ticking time bomb’ for the healthcare sector. This is due to demographic changes such as ageing populations, which are expected to increase the demand for healthcare and social care in the future (WHO,2017, 2022). Further, the WHO highlights the importance of working against inefficiencies and strengthening RNs’ work motivation as strategies to address the shortage of RNs (WHO, 2021, 2022). In the present study, we investigate RNs’ perceptions of the meaningfulness and relevance of their work tasks by using the concept of illegitimate tasks (Semmer et al., 2010). We explore potential associations between the perception of illegitimate tasks and various dimensions of work motivation.

The present study addresses the following knowledge gaps: Few studies in healthcare settings have included RNs’ experiences of illegitimate tasks (e.g., Killponen, 2021; Anskär et al., 2019). As far as we know, none of these studies have explored illegitimate tasks in relation to work motivation within the RN population. Additionally, given the increasing demand for healthcare and resulting ongoing shift to providing more care outside hospitals (European Commission, 2018; WHO, 2017), it is of interest to examine whether there are differences in experienced illegitimate tasks between RNs working in hospital settings and those working in primary care or home healthcare. This has, to the best of our knowledge, not previously been done.

### 1.1 Illegitimate tasks and work motivation

#### 1.1.1 Illegitimate tasks

The construct of illegitimate tasks, developed by Jacobshagen and Semmer, originated from the stress-as-offence-to-self (SOS) theory (Semmer et al., 2010, 2015). The SOS framework is rooted in the idea that individuals aim to uphold a positive self-image, and that threats to this are often at the core of stressful experiences (Semmer et al., 2019). The concept of illegitimate tasks measures to what extent an individual perceives work tasks as relevant and meaningful in relation to their role and identity. What is perceived to be an illegitimate task thus varies between different work or social contexts (Semmer et al., 2015). For instance, in a healthcare setting, documentation is essential for patient safety and is a core task for RNs and other employees. However, if the system or routines are ineffective, or if employees need to perform documentation that they perceive as not medically motivated, they may experience tasks related to this as illegitimate (Anskär et al., 2022; Kilponen et al., 2021). In other words, if an employee does not understand the purpose of a specific task, it can be experienced as illegitimate (Semmer et al., 2019). The construct of illegitimate tasks can be categorised into two subconstructs: unreasonable tasks and unnecessary tasks, which can be analysed separately or as an overarching construct (Semmer et al., 2019). Unreasonable tasks are perceived as falling outside the professional jurisdiction, meaning that they are not included in one's area of responsibility and should be performed by someone else. Unnecessary tasks are perceived as not needing to be carried out at all or not needing to be carried out if things were organised differently (Semmer et al., 2015). In summary, the concept of illegitimate tasks involves examining whether work tasks are perceived as inherently meaningful and/or carried out by the appropriate person or occupation (Semmer et al., 2019). In the present study, we analyse illegitimate tasks as an overarching concept, including the two subconstructs, in relation to work motivation.

#### 1.1.2 Work motivation

Work motivation is a complex and multifaceted concept that can be defined in a variety of ways and from a variety of perspectives (e.g. Gagné and Deci, 2005). Self-determination theory examines how external influences and internal drivers interact. For instance, people can be driven by external rewards or rules without understanding why, or intrinsically motivated by interests, care, curiosity and values. Such intrinsic motivations, though not always externally rewarded, can offer stronger motivation, creativity and endurance (Deci et al., 2017). According to self-determination theory, motivation can be strengthened when, for example, an RN understands the purpose of tasks and why these tasks need to be performed by a professional RN. This understanding might lead the RN to carry out the tasks willingly and with enjoyment (Deci et al., 2017; Deci and Ryan, 2000). Following the logic behind the work motivation theory, illegitimate tasks are likely to be negatively associated with RNs’ work motivation, since illegitimate tasks are work tasks that are perceived as either unreasonable or unnecessary. However, this association has not been the subject of systematic empirical investigation.

In this study, we focused on four dimensions related to work motivation: work engagement, opportunities to provide high-quality care, employer satisfaction and intention to remain at the workplace. Work engagement is characterised by high levels of energy, a sense of significance in one's work challenges, enthusiasm and being deeply immersed in one's work (Ryan and Deci, 2017; Schaufeli et al., 2002, 2019). Providing high-quality care is one of RNs’ core missions. The opportunity to work with core tasks might provide a sense of meaning, which is central to motivation (Amabile and Kramer, 2007; Deci et al., 2017). An employee's experience as regards their employer, for instance satisfaction, is related to work motivation and willingness to remain at the workplace (Amabile and Kramer, 2007; Van den Broeck et al., 2016).

#### 1.1.3 Previous research of illegitimate tasks

The concept illegitimate tasks has been studied in various settings and in relation to a variety of outcomes (Ding and Kuvaas, 2022; Semmer,2019). For instance, it has been found to be associated with negative health and emotional effects, such as lower self-esteem (Sonnentag and Lischetzke, 2018), poorer sleep (Semmer et al., 2015), increased cortisol release (Kottwitz et al., 2013), increased anger and frustration (Eatough et al., 2016), higher incidence of musculoskeletal pain (Kottwitz et al., 2021) and higher risk of burnout (e.g., Ouyang et al., 2022; Semmer et al., 2019). There is also research that focuses specifically on dimensions related to motivation, though not including RNs. For example, in a study conducted in Germany, which involved physicians practicing in general medicine/primary care, it was found that illegitimate tasks were positively associated with burnout and negatively associated with job satisfaction (Werdecker and Esch, 2021). Similarly, an association between a higher degree of illegitimate tasks and decreased job satisfaction was found in a sample of managers from health and social care, education and technical services in a study conducted in Sweden (Björk et al., 2013). Furthermore, associations between illegitimate tasks and increased turnover intention have been found in several occupational groups, such as German information technology professionals (Apostel et al., 2018), managers in various types of municipal services (Cregård and Corin, 2019) and in a study that explored the relationship within a Chinese cultural context, involving a sample of employees from several different occupations (Zeng et al., 2021). Moreover, Omansky et al. (2016) discovered an association between a higher degree of illegitimate tasks and lower intrinsic motivation amongst university employees and students in the US.

There are some studies of illegitimate tasks in various healthcare settings which have included RNs in their samples. In a study of primary healthcare centres in Sweden, 36 % of participants in all staff groups reported a high degree of unnecessary work tasks, and 17 % reported unreasonable work tasks. For instance, a high degree of illegitimate tasks negatively associated with the proportion of self-reported direct patient-related work tasks and positively associated with a self-reported high proportion of organisation-related administration and service work tasks (Anskär et al., 2019). Further, in a study conducted at a municipal healthcare organisation in Finland, with a sample including some RNs, researchers found a significant association between higher levels of illegitimate tasks and lower work engagement (Kilponen et al., 2021). In the same study, they also identified eight different categories of illegitimate tasks that healthcare workers might face. The illegitimate tasks were related to dysfunctional technology, impractical or outdated working habits, unnecessary procedures, tasks related to bureaucratic demands, conflicting or unclear demands, tasks with insufficient resources and with difficult consequences, and tasks outside the occupational role. The last category was the most common amongst RNs in the study (Kilponen et al., 2021). Furthermore, in a study involving a sample of assistant nurses and RNs from Swedish hospitals, the researchers identified a positive correlation between elevated levels of illegitimate tasks and the intention to leave the workplace. Notably, the measurement of illegitimate tasks was conducted using a validated instrument developed by Oxenstiena, which included a single item (Eriksson et al., 2021; Oxenstierna et al., 2008). Moreover, of the participating assistant nurses and RNs in a study from China, 30 % spent some or a lot of time on non-professional tasks, which can be seen as illegitimate, was although this was not measured with the construct illegitimate tasks (Liu et al., 2018).

### 1.2 Differences between workplaces

It is crucial for healthcare sectors to operate efficiently and effectively, regardless of the healthcare setting (WHO,2022). In Sweden and other Western countries, a strategy to cope with demographic changes such as ageing populations and integrated care for older people is to transfer some hospital-based care to primary care or home healthcare (European Commission, 2018; WHO, 2017). This transfer requires that medical staff, including RNs, increasingly choose to and continue to work in primary care and home healthcare. Therefore, it is of interest to acquire more knowledge about how RNs experience illegitimate tasks in primary care or home healthcare, in comparison with RNs employed in hospitals.

In summary, while there is previous research on illegitimate tasks in the healthcare sector, there are – to the best of our knowledge – few studies specifically focused on RNs. In particular, there is a lack of studies focusing on the association between illegitimate tasks and dimensions of work motivation. Moreover, we have not been able to find any study of whether RNs’ perceptions of illegitimate work tasks vary between different workplaces, or if associations between illegitimate tasks and work motivation differ between workplaces.

### 1.3 Aim and research questions

Our overall aim was to explore the association between RNs' experiences of illegitimate tasks and work motivation operationalised as four dimensions: work engagement, opportunities to provide high-quality care, employer satisfaction and intention to remain at the workplace. To address this aim, three specific research questions were asked: (1) Is there an association between illegitimate tasks and work motivation? (2) Do the levels of reported illegitimate tasks differ between RNs working in hospitals and those working in primary care or home healthcare settings? (3) Do associations between illegitimate work tasks and work motivation differ with type of workplace?

## 2 Methods

### 2.1 Study design and population

We used a cross-sectional design and data from the Longitudinal Occupational Health Survey in Healthcare Sweden for 2022. The questionnaire development has been described elsewhere (Hagqvist et al., et.al,2022). An invitation to participate in the survey was distributed by Statistics Sweden in March 2022 to May 2022, including information for accessing an electronic questionnaire. Up to three reminders to participate were sent out. In the second reminder, participants also received a paper version of the questionnaire (response rate 37.3 %). The Longitudinal Occupational Health Survey in Healthcare Sweden cohort included a representative sample of physicians, RNs and nurse assistants. In this study, we used data for the RNs. To work as an RN in the Swedish healthcare system, a person must have a bachelor's degree in nursing or medicine and obtain a license. The population was RNs in Sweden between 18 and 69 years old (*N* = 109,475). Statistics Sweden drew the sample (*n* = 8000) from the Swedish Occupational Register in 2020 (nurse code SSYK ‘222′, ’223’) and from the Register on Participation in Education in 2020–2021. People who have not been working as RNs in Sweden in the preceding year were removed from the sample. To get a geographical spread, the population was stratified based on administrative healthcare regions. For the purpose of the present study, we restricted the sample to RNs who reported working in either a hospital, primary care or home healthcare. Thus, we excluded school nurses, RNs on occupational healthcare and RNs who indicated more than one workplace.

### 2.2 Measures

#### 2.2.1 Illegitimate tasks

Illegitimate tasks were measured using the Bern Illegitimate Tasks Scale (Semmer et al., 2015). Illegitimate tasks can be analysed by utilising the two subconstructs – unreasonable tasks and unnecessary tasks – separately or as an overarching construct. Currently, no clear patterns of differentiated associations seem to exist (Semmer et al., 2019). Since the main focus of this study was whether RNs' experiences of illegitimate tasks were associated with the four dimensions of work motivation, not whether there were any differences between unreasonable and unnecessary tasks in this regard, illegitimate tasks were measured using the overarching construct (consisting of eight items). The first four items were introduced with the lead-in phrase ‘Do you have work tasks to take care of, which you believe …’, followed by the statements ‘should be done by someone else?’, ‘are going too far, which should not be expected from you?’, ‘put you into an awkward position?’ and ‘are unfair that you have to deal with?’. The second four items were introduced with the lead-in phrase ‘Do you have work tasks to take care of, which keep you wondering if …’, followed by the statements ‘they have to be done at all?’, ‘they make sense at all?’, ’they would not exist or could be done with less effort, if things were organised differently?’ and ‘they would not exist or could be done with less effort, if other people made less mistakes?’. Responses are given on a five-point Likert scale from ‘never’ to ‘frequently’. Higher scores corresponded to a higher degree of perceived illegitimacy for the work tasks. In the present study, a mean sum score was used for the illegitimate task index, including all eight items, with an internal consistency of α = 0.85.

#### 2.2.2 Dimensions related to work motivation

Work motivation was measured based on four dimensions: work engagement, opportunities to provide high-quality care, employer satisfaction and intention to remain at the workplace.

*Work engagement* was measured using the Ultra-Short Measure for Work Engagement, a validation scale also used in the new Swedish standard version of Copenhagen Psychosocial Questionnaire Version III (Berthelsen et al., 2020; Schaufeli et al., 2019). The Copenhagen Psychosocial Questionnaire is a widely used instrument and covers a broad range of domains, with work engagement being part of the Work-Individual Interface (Burr et al., 2019)*.* The new Swedish standard version of the Copenhagen Psychosocial Questionnaire III was developed for use at workplaces and research in Swedish (Berthelsen et al., 2020; Burr et al., 2019). The questionnaire consists of three subscales in one index. Questions related to work engagement are introduced with the lead-in phrase ‘How often do you experience the following? At my work …’ followed by the statements ‘I feel bursting with energy when I perform my work’, ‘I am enthusiastic about my work’ and ‘I am strongly engaged in my work’. Responses are given on a five-point Likert scale from ‘never/almost never’ to ‘always’. Internal consistency was 0.78. A mean sum score was used for the work engagement index.

*Opportunities to provide high-quality care* of patients was measured with a single item, from the Longitudinal Occupational Health Survey in Healthcare Sweden (author): ‘At my workplace, I have the opportunity to provide all patients with high-quality care.’ Responses were given on a five-point Likert scale from ‘to a very low extent’ to ‘to a very high extent’.

*Employer satisfaction* was measured with a single item, from the Longitudinal Occupational Health Survey in Healthcare Sweden (author): ‘How satisfied or dissatisfied are you with your employer?’. Responses were given on a five-point Likert scale from ‘very dissatisfied’ to ‘very satisfied’.

*Intention to remain at the workplace* was measured with a single item asking ‘How often in the last 12 months have you thought of applying for a new job?’ with responses given on a five-point scale: 1 = every day; 2 = a few times a week; 3 = a few times a month; 4 = a few times in the last 12 months; 5 = not in the last 12 months. The item has been used in the Swedish Longitudinal Occupational Survey of Health as well as in the Longitudinal Occupational Health Survey in Healthcare Sweden (authors; Hanson et al., 2018). Since the distances between the response options were not equal, we decided to dichotomise the item (Field, 2018). The dichotomous variable was defined as follows: (1) Has thought of applying for a new job in the last 12 months (1–4) (reference); (2) Has not thought of applying for a new job in the last 12 months (5).

#### 2.2.3 Workplaces

Type of workplace was analysed in three groups: (1) hospital, (2) primary care and (3) home healthcare. The group hospital included RNs who reported they worked at a hospital with inpatient care. The primary care group included RNs who reported they worked in a primary healthcare centre or in a specialised outpatient clinic. Home healthcare included RNs who reported they worked with providing healthcare in patients’ own homes. The primary group of patients who need home healthcare are elderly people in need of care and support. These patients typically reside either in their own home or in a home-like environment such as a nursing home. The workplace variable was analysed as a categorical variable and hospital was set as the reference.

#### 2.2.4 Covariates

We adjusted for sex (male/female), age (categorised as 30 years or less, 31–40 years, 41–50 years, 51–60 years or 61 years or over), years working as a RN (categorised as less than 5 years, 10–15 years or more than 15 years), specialist education (yes or no) and leadership assignments (yes or no) (Table 1). In addition, we adjusted for the workplace variable (hospital, primary care or home healthcare) in the analyses of associations between illegitimate tasks and the dimensions related to work motivation (Tables 3 and 4).

**Table. 1:** Descriptive statistics of sample stratified by type of workplace (*n* = 2333).

Variable		Total sample without calibrated weights % (*n*)	Total sample with calibrated weights%*	RN within hospital%*	RN within primary care%*	RN within home healthcare%*	Statistical significance between types of workplace, chi-squared*
Sex	male	9.7 *(227*)	11.1	14.1	4.7	7.8	<0.001
	female	90.3 (*2106*)	88.9	85.9	95.3	92.2	
Age (years)	≤ 30	13.0 (*304*)	14.8	20.0	4.5	8.7	<0.001
	31–40	24.3 (*568*)	26.8	28.4	24.3	23.7	
	41–50	21.5 (*502*)	22.2	20.4	26.5	23.4	
	51–60	23.4 (*547*)	21.3	18.3	26.5	25.9	
	> 61	17.7 (*412*)	14.9	12.9	18.1	18.4	
Years as an RN	< 5	18.5 (*431)*	20.6	25.3	9.3	18.4	<0.001
	5–10	14.9 *(347)*	16.6	19.0	11.8	13.8	
	10–15	15.3 *(356)*	15.8	14.0	20.9	15.6	
	> 15	51.2 *(1195)*	47.0	41.7	58.0	52.2	
	missing	.2 *(4)*					
Specialist education	No	47.4 *(1107)*	49.3	53.1	29.7	65.9	<0.001
	Yes	52.0 *(1213)*	50.7	46.9	70.3	34.1	
	missing	.6 *(13*)					
Leadership assignment	Yes	9.0 (*210)*	9.0	9.7	8.5	6.9	ns.
	No	91.0 (*2123)*	91.0	90.3	91.4	93.1	

Note. ns = not statistically significant.

⁎ calibrating weights were used.

As it cannot be theoretically ruled out that a person's experience of illegitimate tasks as well as their experience of work motivation is affected by their general life satisfaction, we included this as an additional control variable in the last model in all regression analyses. RNs’ experiences of general life satisfaction were measured with a single item: ‘In general, how satisfied are you with your life?’ Responses were given on an eight-point Likert scale from ‘very dissatisfied’ to ‘very satisfied’.

### 2.3 Analytical strategy

Data were analysed with SPSS version 28.

Weights were applied to minimise the non-response error in all analyses. Calibrating weights were calculated by Statistics Sweden in accordance with the description of Särndal and Lundström (2005). Calibrating weights yield better generalisability of the findings. To present the characteristics of the sample, we used descriptive statistics (frequencies and percentages), stratified by type of workplace. Differences between groups were estimated using a chi-squared analysis, as shown in Table 1. The specific questions/items in the illegitimate task scale and the distributions of the response options are presented in Table 2.

**Table 2:** Distribution of responses for the eight items in the illegitimate task index (*n* = 2333).

	Number of responses (%)		Never%	Rarely%	Sometime%	Frequently%	Very frequently%	Mean Likert scale score
Do you have work tasks to take care of, which you believe.								
…should be done by someone else?	2325	(99.7)	3.1	12.9	35.4	29.2	19	3.48
…are going too far, which should not be expected from you?	2317	(99.3)	6.3	25.1	32.8	21.6	13.5	3.11
…put you into an awkward position?	2322	(99.5)	9.7	39.3	35.5	10.9	4.0	2.60
…are unfair that you have to deal with?	2319	(99.4)	11.3	38.6	31.7	12.7	5.1	2.61
Do you have work tasks to take care of, which keep you wondering if …								
…they have to be done at all?	2320	(99.4)	9.4	33.1	36.0	15.0	5.9	2.75
…they make sense at all?	2321	(99.5)	6.4	25.1	33.1	21.6	13.2	3.10
…they would not exist or could be done with less effort, if things were organised differently?	2313	(99.1)	2.6	14.9	37.1	28.1	16.4	3.41
…they would not exist or could be done with less effort, if other people made less mistakes?	2317	(99.3)	10.6	32.8	33.9	15.0	7.0	2.75

Linear regression analysis was used to estimate the relationship between illegitimate tasks and three outcomes: work engagement, opportunities to provide high-quality care and employer satisfaction (Table 3). We first analysed the construct illegitimate tasks in a crude model. Next, in Model 1, we adjusted for workplace, sex, age, years as a RN, specialist nursing education and leadership assignments. In Model 2, we also adjusted for general life satisfaction. In the final step of the regression analysis, we added an interaction term in order to estimate if associations between illegitimate tasks and work motivation differed by type of workplace (research question 3).

**Table 3:** Associations between RNs’ experiences of illegitimate tasks (independent variable) and RNs’ experiences of the three dependant variables: work engagement, opportunities to provide high-quality care, employer satisfaction. Linear regression analyses using calibrating weights (work engagement, *n* = 2257, opportunities to provide high-quality care, *n* = 2246, and employer satisfaction, *n* = 2266).

	Crude model			Model 1		Model 2	
	b (SE)	CI 95 %		b (SE)	CI 95 %	b (SE)	CI 95 %
**Work engagement**							
**Illegitimate tasks**	-0.26 (0.02)	-0.30; -0.23	-0.21 (0.02)⁎⁎		-0.25; -17	-0.14 (0.02)⁎⁎	-0.18; -0.10
*R2 adjusted*	.08		.11			.19	
**Opportunities to provide high-quality care**							
**Illegitimate tasks**	-0.59 (0.03)**	-0.64; -0.54	-0.51 (0.03)⁎⁎		-0.56; -0.45	-0.46 (0.03)⁎⁎	-0.51; -0.40
*R2 adjusted*	.19		.24			.26	
**Employer satisfaction**							
**Illegitimate tasks**	-0.66 (0.03)**	-0.72; -0.60	-0.66 (0.03)⁎⁎		-0.73; -0.60	-0.60 (0.03)⁎⁎	-0.67; -0.54
*R2 adjusted*	.17^6^		.19			.20	

**Note.** beta coefficient (b), std. error (SE) in parenthesis, and confidence interval (CI) 95 %.

⁎⁎ *p* < 0.001. Crude model: Illegitimate tasks. Model 1: Crude + workplace, sex, age, years as a RN, specialist nursing education, leadership assignments as control variables. Model 2: Model 1 + general life satisfaction.

Binary logistic regression was used to predict the relationship between illegitimate tasks and the outcome intention to remain at the workplace. The analytical procedure was the same as in the linear regression above (Table 4).

**Table. 4:** Associations between RNs’ experiences of illegitimate tasks and the dependant variable RNs’ intention to remain at their workplace. *n* = 2253. Binary logistic regression using calibrating weights.

Intention to remain at the workplace						
	Crude model		Model 1		Model 2	
	OR	Cl 95 %	OR	Cl 95 %	OR	Cl 95 %
**Illegitimate tasks**	.26⁎⁎	.22; 0.31	.29⁎⁎	.24; 0.34	.32⁎⁎	.27; 0.38
Overall%	77⁎⁎		77⁎⁎		78⁎⁎	

**Note.** OR = adjusted odds ratio, CI = confidence interval.

⁎⁎ *p* < 0.001. Crude model: Illegitimate tasks. Model 1: Crude + workplace, sex, age, years as a RN, specialist nursing education, leadership assignments as control variables. Model 2: Model 1 + general life satisfaction.

To analyse if levels of reported illegitimate tasks differed between RNs working at hospitals and RNs employed in either primary care or home healthcare, we used linear regression. The three categories were hospital, primary care and home healthcare. Hospital was used as the reference. First, as a crude model, we analysed the three categories: hospital (reference), primary care and home healthcare. In Model 1, we adjusted for sex, age, years as a RN, specialist nursing education and leadership assignments. Lastly, in Model 2, we adjusted for general life satisfaction as well (Table 5).

**Table. 5:** Association between working in hospitals (ref), primary care or home healthcare, and levels of illegitimate tasks reported. *n* = 2263. Linear regression analysis using calibrating weights.

		Crude model		Model 1		Model 2	
	b (SE)		CI 95 %	b (SE)	CI 95 %	b (SE)	CI 95 %
Primary care	-0.18 (0.04)⁎⁎		-0.25; -0.11	- 0.08 (0.04)*	- 0.15; -0.003	-0.06 (0.04)	ns
Home healthcare	.10 (0.04)*		.02; 0.19	.15 (0.04)⁎⁎	.06; 0.24	.16 (0.04)⁎⁎	.07; 0.24
R2 adjusted	.02			.09		.15	

Note. Beta coefficient (b), std. error (SE) in parenthesis, and confidence interval (CI), 95 %.

ns = not statistically significant.

⁎ *p* < .05.

⁎⁎ *p* < .001. Crude model: Workplace: hospital (ref), primary care or home healthcare. Model 1: Crude model + sex, age, years as a RN, specialist nursing education, leadership assignments as control variables. Model 2: Model 1 + general life satisfaction.

### 2.4 Ethical considerations

The study was approved by the Swedish Ethical Review Authority (Dnr: 2022-03480-01; 2022-00310-02) and followed the ethical standards as described in the Declaration of Helsinki (World Medical Association, 2013).

## 3 Results

The results section is organised as follows: First, we present descriptive data on participating RNs and overall experiences of illegitimate tasks. This is followed by an analysis of RNs’ experiences of illegitimate tasks and their associations with the four dimensions related to work motivation. Lastly, we present differences in experiences of illegitimate tasks between RNs working in hospitals, primary care or home healthcare, respectively.

### 3.1 Descriptive statistics of demographic and illegitimate task

#### 3.1.1 Demographic of RNs

The sample was RNs in Sweden between 23 and 69 years old, who had worked as RNs in a hospital, primary care or home healthcare in the preceding year (*n* = 2333). Respondents, stratified by type of workplace, are presented in Table 1. It can be noted that amongst RNs in hospitals, 48.4 % were 40 years old or younger, compared with 28.8 % of RNs in primary care and 32.2 % of RNs in home healthcare. Of the RNs in primary care, 70.3 % had a specialist education (one year of studies after bachelor's degree), compared with 46.9 % of RNs in hospitals and 34.1 % of RNs in home healthcare (Table 1).

#### 3.1.2 Descriptive statistics of RNs’ experiences of illegitimate tasks

The overall index for the concept of illegitimate tasks shows that 25 % of RNs often or very often experienced illegitimate tasks. Further, 48.2 % of RNs reported that they frequently or very frequently (mean Likert scale score 3.48) had tasks that should be done by someone else. Furthermore, 44.5 % (of RNs reported that they frequently or very frequently (mean Likert scale score 3.41) had tasks that would not need to exist if the organisation were structured differently (Table 2).

### 3.2 Associations between illegitimate tasks and work motivation dimensions

#### 3.2.1 Work engagement, opportunities to provide high-quality care and employer satisfaction

There were statistically significant associations between reporting higher levels of illegitimate tasks and reporting lower levels of work engagement, opportunities to provide high-quality care and employer satisfaction in all models.

A 1-step increase of the illegitimate task scale predicted a 0.26-step decrease in the RNs’ work engagement in the crude model and a 0.14-step decrease in the fully adjusted model. Further, each 1-step increase in illegitimate tasks was associated with a 0.59-step decrease in RNs’ opportunities to provide high-quality care in the crude model and a 0.46-step decrease in the fully adjusted model. Lastly, a 1-step increase in the perception of illegitimate tasks predicted a 0.66-step decrease in RNs’ satisfaction with their employer in the crude model and a 0.60-step decrease in the fully adjusted model (Table 3). There was no indication of an interaction effect between illegitimate work tasks and type of workplace in relation to work motivation.

#### 3.2.2 Intention to remain at the workplace

As shown in Table 4, there was a statistically significant negative association between RNs’ experiences of higher level of illegitimate task and their intention to remain at the workplace. In the fully adjusted model, the odds ratio (OR) to remain at the workplace was 0.32 (95 % CI: 0.27; 0.38). There was no indication that the association differed with type of workplace.

### 3.3 Differences between experiences of illegitimate tasks by RN workplace

Compared with RNs who worked in hospitals, RNs in primary care reported lower levels of illegitimate tasks in the crude model and in Model 1. However, when we adjusted for RNs’ general life satisfaction, the association was no longer statistically significant (Table 5, Model 2). RNs who worked in home healthcare reported higher levels of illegitimate tasks than RNs who worked in hospitals, in all statistical models (Table 4).

## 4 Discussion

This study sought to explore if RNs’ perceived levels of illegitimate tasks were associated with dimensions related to work motivation, if levels of reported illegitimate tasks differed between RNs working in hospitals, primary care or home healthcare, and if associations between illegitimate tasks and work motivation differed with type of workplace. The key findings highlight a statistically significant association between higher levels of illegitimate tasks and lower levels of work engagement, lower opportunities to provide high-quality care, lower employer satisfaction and lower intention to remain at the workplace. Furthermore, RNs working in home healthcare reported higher levels of illegitimate tasks than RNs working in hospitals. There was no indication that associations between illegitimate tasks and work motivation differed depending on type of workplace.

### 4.1 Illegitimate tasks and work motivation

Overall, the result indicated that one fourth of RNs frequently had illegitimate tasks. RNs frequently or very frequently had tasks they believed that others could do (48.2 %) and tasks that would not need to exist if things were organised differently (44.5 %). Below, we will discuss possible consequences of illegitimate task for work motivation in terms of work engagement, opportunities to provide high-quality care, employer satisfaction and the intention to remain at the workplace.

#### 4.1.1 Work engagement

Our results indicated that there was an association between higher levels of illegitimate tasks and RNs’ experiences of work engagement. Work engagement is about feeling engaged and interested, gaining energy from one's work, something closely related to intrinsic motivation (Deci et al., 2017). Our findings reinforce the idea that illegitimate tasks counteract intrinsic motivation as described in self-determination theory (Deci et al., 2017). Furthermore, the results strength those of previous research in healthcare settings, even amongst other employees than RNs, for instance healthcare workers in a Finnish healthcare organisation (Kilponen et al., 2021) and first-line managers in Sweden (Björk et al., 2013). It also appears that the relationship between work engagement and illegitimate tasks does not vary significantly across different workplace settings, including hospitals, primary care and home healthcare, as the interaction analysis did not show statistically significant results. This underscores the importance of minimising the occurrence of illegitimate tasks irrespective of workplace. The association between a higher level of illegitimate tasks and RNs' experiences of work engagement is important in the context of enhancing work motivation. A potential improvement could lead to more RNs remaining in the workplace, addressing the issue of the shortage of RNs (WHO, 2021, 2022).

#### 4.1.2 Providing high-quality care

Our findings suggested that RNs experiencing higher level of illegitimate tasks reported lower opportunities to provide high-quality care than those with lower levels of illegitimate tasks. According to Kilponen et al. (2021), the most common illegitimate tasks amongst RNs are those that fall outside their professional jurisdiction. Additionally, a previous qualitative study has shown that RNs who experience illegitimate tasks may not always have sufficient time for necessary tasks (Anskär et al., 2022). This may be significant, as lost opportunities to provide good care can decrease patient safety, which was highlighted in a study conducted in a US nursing home (White et al., 2019). From an RN motivational perspective, fewer illegitimate tasks may create opportunities for RNs to perform central and meaningful tasks, which could potentially increase their work motivation (Ahlstedt et al., 2019; Deci et al., 2017). Furthermore, it is important to emphasise that the workplace's influence seems to be less significant, as our research findings suggest that the correlation between illegitimate tasks and the opportunity to provide high-quality care remains consistent across different workplace settings, including hospitals, primary care and home healthcare.

#### 4.1.3 Employer satisfaction and intention to remain at the workplace

Our results also indicated associations between RNs’ experiences of higher levels of illegitimate tasks and lower levels of employer satisfaction. This finding aligns with the fact that RNs commonly perceived illegitimate tasks as being connected to the organisational structure. For instance, the two items with highest reported frequency were work tasks which RNs believed should be done by someone else and work tasks that would not exist or could be done with less effort if things were organised differently (Table 2). In other words, according to self-determination theory (Deci et al., 2017), RNs experienced illegitimate tasks as arising from organisational reasons rather than, for instance, patients’ needs. This might lead to a decrease in motivation over time, as such tasks would not reinforce intrinsic motivation through interests, care and personal values. Furthermore, our findings indicated a negative association between RNs' experiences of higher levels of illegitimate tasks and their intention to remain at the workplace. The results from our study complement previous findings of associations between illegitimate tasks and employees’ turnover intention from other sectors, for instance amongst information technology professionals (Apostel et al., 2018) and public sector managers in Sweden (Cregård and Corin, 2019).

#### 4.1.4 Practical implications

Lower levels of illegitimate tasks in daily work may increase perceived meaningfulness and might strengthen RNs’ work motivation (Deci et al., 2017; Deci and Ryan, 2000). Based on the results from this study, as well as previous studies (e.g., Eriksson et al., 2021), it could be argued that allocating more time for crucial RN duties would enhance the profession's attractiveness, an aspect that the WHO also considers significant to motivation (WHO, 2021). Given that the perception of what constitutes an illegitimate task can vary across different contexts or social meanings (Semmer et al., 2015), it is recommended that managers and organisations engage in dialogue with RNs to identify and address illegitimate tasks perceived in their daily work. This dialogue should focus on RNs’ work motivation and willingness to remain in the workplace, rather than solely on cost-effectiveness.

### 4.2 Impact of workplace

In Western countries, responsibilities are being transferred from hospital-based care to primary care or home healthcare (European Commission, 2018; WHO, 2022). Therefore, we investigated whether levels of reported illegitimate tasks differed between RNs working in hospitals and RNs who worked in either primary care or home healthcare. The results indicated that RNs who worked in home healthcare experienced higher levels of illegitimate tasks than RNs employed in hospitals. Home healthcare is particularly important for the elderly, making these results important in light of current demographic changes with ageing populations and increasing occurrence of chronic diseases and multimorbidity in many countries (WHO, 2017). On the other hand, our results indicated that RNs in primary care experienced lower levels of illegitimate tasks than RNs who worked in hospitals, though this result was not statistically significant when we adjusted for general satisfaction with life. Still, there might be potential for improvement in primary care. For instance, previous studies from primary care centres show that some administrative tasks, such as double documentation, are experienced as illegitimate (Anskär et al., 2022, 2019). It is noteworthy that the results indicated that the association between illegitimate tasks and dimensions related to motivation was valid across workplaces. Therefore, it is equally important for all organisations and managers to pay attention to levels of perceived illegitimate tasks, regardless of the workplace. Considering the global goal of providing more care outside hospitals, it is important to increase knowledge through further research into illegitimate tasks in different workplaces and healthcare settings.

### 4.3 Strengths and limitations

There are some limitations in this study that should be addressed. First, the study's cross-sectional design limits our ability to draw conclusions about causality between the variables of interest. Furthermore, as self-reported data were used for both predictor and outcome variables, we cannot rule out the possibility that the results indicate stronger associations between the variables than those that exist in reality. In an attempt to account for negative affectivity and thereby to some extent decrease the risk of common method bias, we included the RNs’ reports of general life satisfaction in the fully adjusted models. Third, our sample consisted only of RNs in Swedish healthcare and the results might not be applicable to healthcare organisations in other countries. There are also some strengths to highlight. First, an important strength is the use of a large, stratified, and nationally representative sample of RNs in Sweden. Furthermore, dropouts are commonly regarded as a factor that introduces uncertainty into statistical analyses. In this study, the use of calibrated weights decrease the impact of such an uncertainty. The generalizability of the results to registered nurses in Sweden and the robustness of the comparisons between RNs working in different healthcare settings are also strengthened.

## 5 Conclusions

This study revealed statistically significant associations between RNs’ experiences of illegitimate tasks and lower levels of four dimensions related to work motivation: work engagement, opportunity to provide high-quality care, employer satisfaction and intention to remain at the workplace. Our results highlighted the importance of reducing the experience of illegitimate tasks in healthcare, as this might reduce RN turnover. Furthermore, the results indicated that RNs in home healthcare experienced more illegitimate tasks than RNs who worked in hospitals. Organisations and managers should take note of this, as more patients may receive healthcare at home or in home-like environments such as nursing homes in the future.

## Funding sources

This research did not receive any specific grant from funding agencies in the public, commercial, or not-for-profit sectors.

## CRediT authorship contribution statement

**Carina Ahlstedt:** Conceptualization, Methodology, Formal analysis, Writing – original draft, Writing – review & editing. **Linda Moberg:** Conceptualization, Methodology, Writing – review & editing, Supervision. **Emma Brulin:** Data curation, Methodology, Writing – review & editing. **Anna Nyberg:** Conceptualization, Methodology, Writing – review & editing, Supervision.

## Declaration of Competing Interest

The authors declare that they have no known competing financial interests or personal relationships that could have appeared to influence the work reported in this paper.
